# Supplementary figures and images for: Power analysis of transcriptome-wide association study: Implications for practical protocol choice
Source: PLoS Genet. 2021 Feb 26;17(2):e1009405. doi: 10.1371/journal.pgen.1009405 (PMC7946362; doi:10.1371/journal.pgen.1009405)

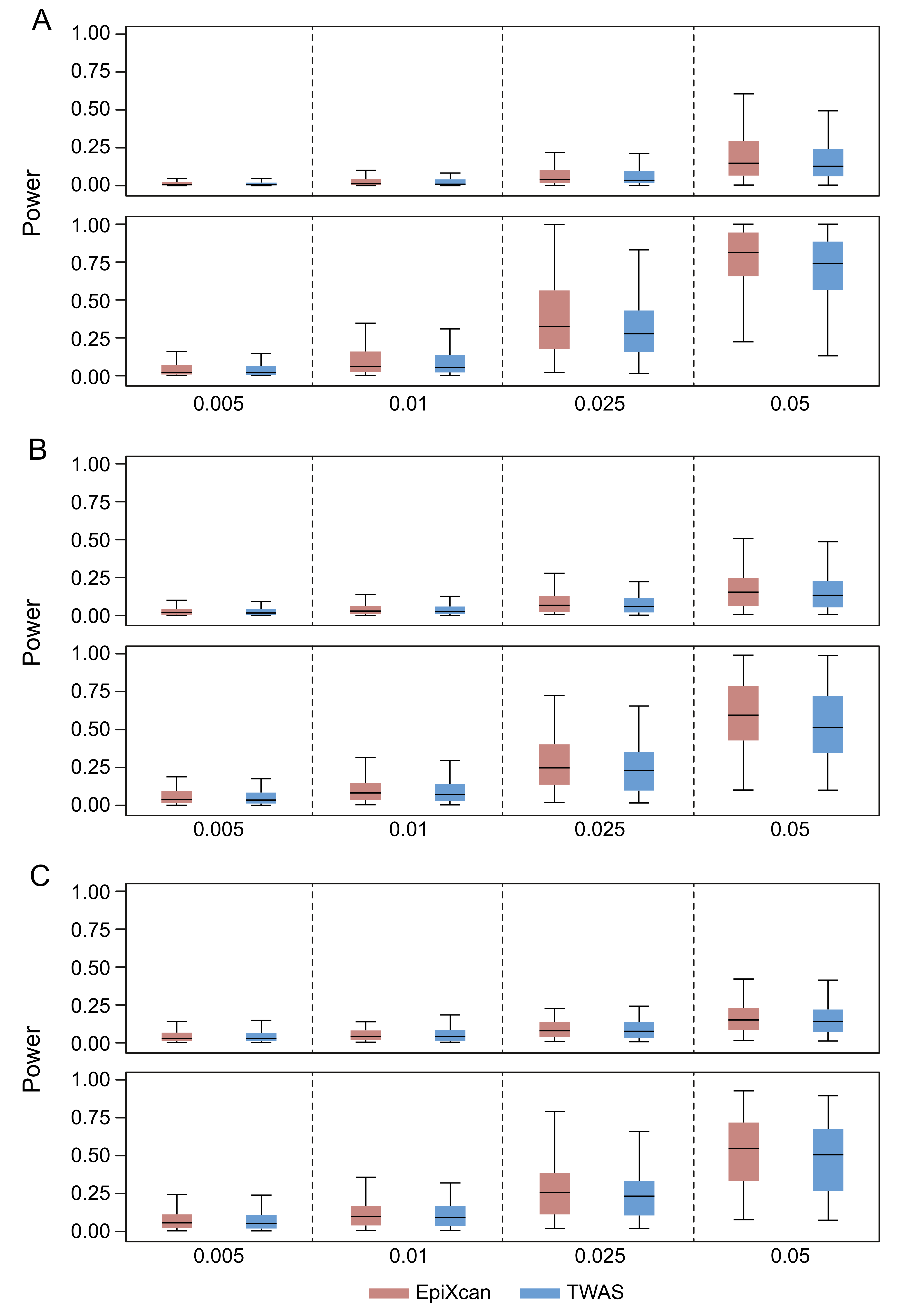

Supplement: S1 Fig — The PVX (phenotypic variance explained by expression) is 0.005, 0.01, 0.025, and 0.05 in the four columns as indicated by the X-axis labels. In each of (a), (b), and (c), the expression heritability for the top and bottom rows are 0.1 and 0.3 respectively. The number of genes contributing to phenotype for (a), (b) and (c) are 4, 9, and 13 respectively. The number of causal variants per gene is randomly sampled from the interval [4,9]. (TIFF) [file pgen.1009405.s001.tiff]

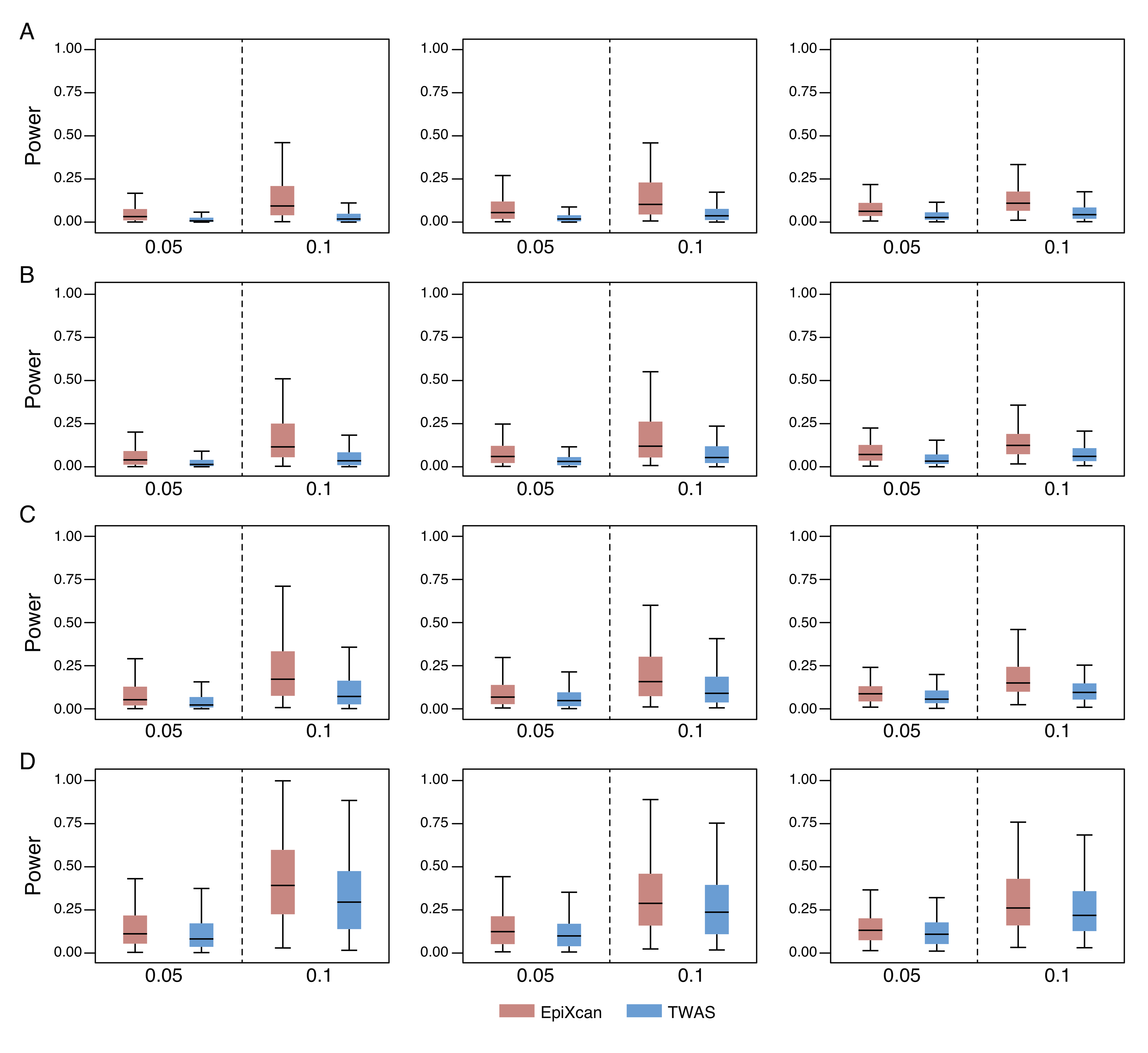

Supplement: S2 Fig — In each panel, the PVX is 0.05 and 0.1 in the left and right columns as indicated by the X-axis labels. In each of (a), (b), (c), and (d), the numbers of genes contributing to phenotype for the left, center, and right panels are 4, 9, and 13 respectively. The expression heritability levels in (a), (b), (c), and (d) are 0.025, 0.03, 0.04, and 0.08 respectively. The number of causal variants per gene is randomly sampled from the interval [4,9]. (TIFF) [file pgen.1009405.s002.tiff]

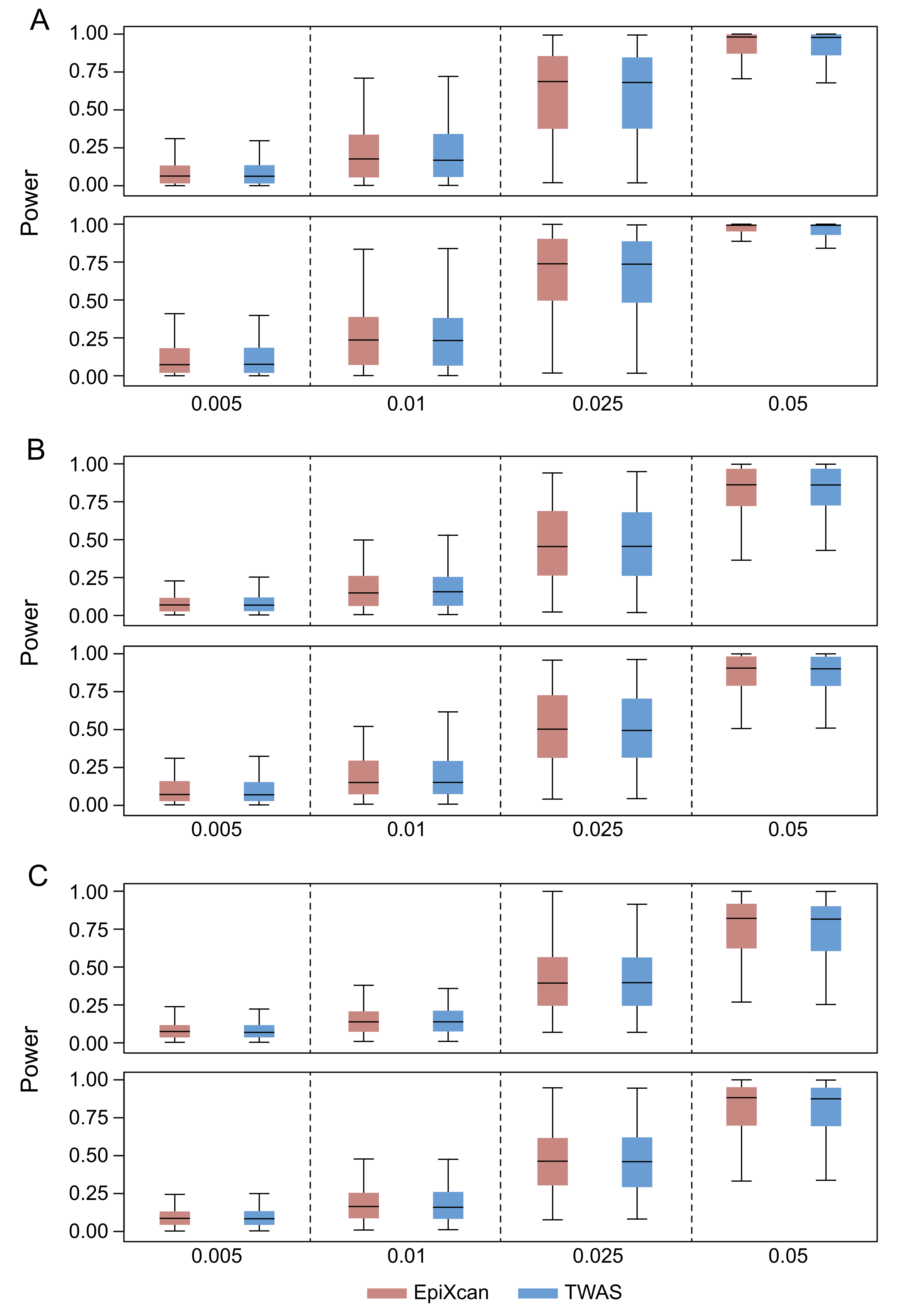

Supplement: S3 Fig — The trait heritability is 0.005, 0.01, 0.025, and 0.05 in the four columns as indicated by the X-axis labels. In each of (a), (b), and (c), the expression heritability for the top and bottom panels are 0.1 and 0.3 respectively. The numbers of genes contributing to phenotype for (a), (b), and (c) are 4, 9, and 13 respectively. The number of causal variants per gene is randomly sampled from the interval [4,9]. (TIFF) [file pgen.1009405.s003.tiff]

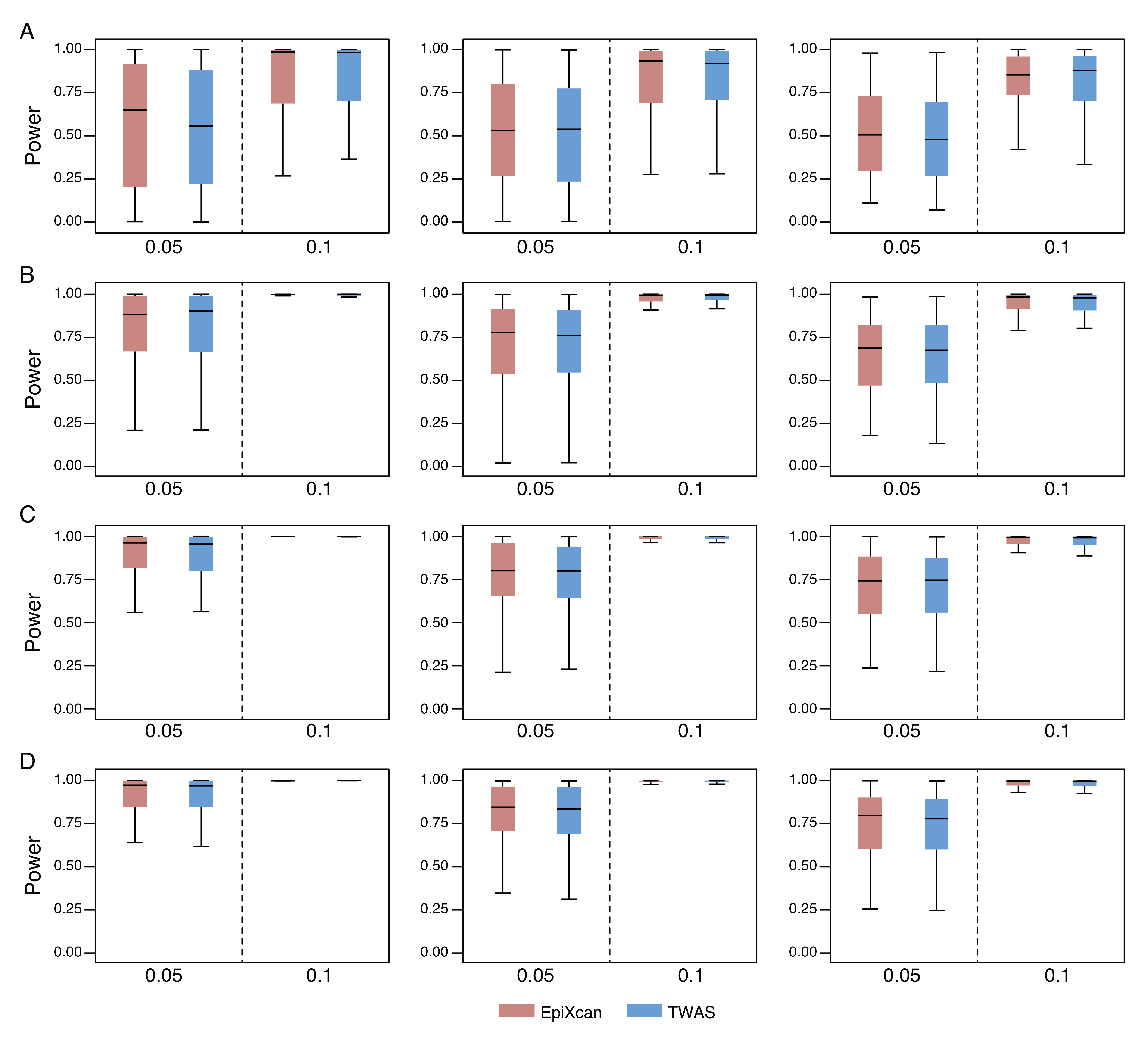

Supplement: S4 Fig — The PVX is 0.05 and 0.1 in the two columns as indicated by the X-axis labels. In each of (a), (b), (c), and (d), the number of genes contributing to phenotype for the left, center, and right panels are 4, 9, and 13 respectively. The expression heritability levels in (a), (b), (c), and (d) are 0.025, 0.04, 0.06, and 0.08 respectively. The number of causal variants per gene is randomly sampled from the interval [4,9]. (TIFF) [file pgen.1009405.s004.tiff]
