# Supplementary material for: Power analysis of transcriptome-wide association study: Implications for practical protocol choice
Source: PLoS Genet. 2021 Feb 26;17(2):e1009405. doi: 10.1371/journal.pgen.1009405 (PMC7946362; doi:10.1371/journal.pgen.1009405)
Supplement: Attachment — Submitted filename: ResponseLetter_Jan11.pdf [file pgen.1009405.s007.pdf]

We would like to see some detailed comparisons with other methods (Epixcan and SMR, reviewer 1) and address the independence of the outcome model and expression model (reviewer 2).

Thank you so much for the opportunity to revise. We have conducted thorough analysis to address the comments from Reviewer #1 and added/changed descriptions based on the comments from Review #2. Please see our point-to-point response below.

Reviewer #1:

In this article, authors provided power analysis of TWAS to indicate practical protocol choice. It is a very interesting topic and the results are pretty interesting. PrediXcan and EpiXcan are cited and the methods performed TWAS to identify trait-associated transcriptomes.

Thank you very much for the positive evaluation.

Just one minor suggestion:

can authors compare Epixcan and the other related methods? Maybe another interesting paper using SMR could provide more insights: DOI: 10.1016/j.ajhg.2017.04.016. PMID: 28552197

Thank you for directing us to EpiXcan. This comment actually provides an excellent opportunity for us to show how to generalize our framework to evaluate the power gain of other protocols!

We have then conducted the analysis to evaluate the power of EpiXcan. We first conduct simulations where a subset of SNPs are assigned increased effects, which reflects the main insight of the EpiXcan paper that epigenetic-relevant functional SNPs have higher impact on variation in gene expression. In particular, we assume the real effect size follows a standard normal distribution N(0,1), and sample effect sizes from this distribution. Assuming these functional SNPs are known (based on various techniques of annotating SNP functions), we relieve their penalty in training the predictive model. Using the predicted expressions, we calculate power using our derived NCP, and compare the resulting analysis with the standard TWAS protocol. Supplementary Fig. S1-S4 depict this quantitative evaluation of the improvement in power due to the contribution of epigenetic-relevant functional SNPs. Evidently, under the causality model EpiXcan indeed increases power by improving expression predictions, although the improvement is more pronounced in the cases that expression heritability is low (Supplementary Fig. S1, S2). However, under the pleiotropy model, EpiXcan only shows almost no increase in power over TWAS (Supplementary Fig. S3, S4). This observation suggests that when DNA mutations contribute to phenotype directly, the benefit of more accurate predictions for expressions may not be substantial.

We have added a subsection in Results to describe the above analysis.

We are also grateful to the mention of the SMR which may also be assessed using similar approaches. However, we are afraid that a thorough analysis of the power of SMR may be out of the scope of this work. Our work is based on closed-form derivations to test power (which is free from different details of implementations), so the derivation of the power of SMR requires lots of other theoretical analysis of the mathematical formulation of SMR. The main focus of this work is TWAS/GWAS/emGWAS/ which focuses on the association between genotype and

phenotype; however SMR aims to identify the related genes and tissues given genotype-phenotype association, which is a different goal and employs completely different statistical models. As such, we respectively discussed this issue and the literature mentioned by the reviewer in Discussion, leaving the in-depth investigation to future work.

Reviewer #2:

In this manuscript, Ding and colleagues studies power of transcriptome-wide association studies (TWAS), providing practical guidelines based on results accordingly. Specifically, the authors compared power of TWAS with two alternative strategies: genome-wide association studies (GWAS) and association between measured gene expression levels with phenotype of interest (termed emGWAS by the authors). The comparisons are meaningful and the consideration of the emGWAS is thoughtful as such data may become increasingly available in the near future. The authors considered two main scenarios: causal or mediation scenario where genetic variants exert effect on phenotype via gene expression, and pleiotropic scenario where genetic variants simultaneously influence both phenotype and gene expression. There are many merits of the study, including analytical derivations to lay the theoretical foundation of power calculations, and carefully designed simulation studies to evaluate the power of TWAS and related approaches, which have clear implications in practice for real studies with or without measured expression data.

Generally speaking, the manuscript is well written with clearly presented methods and results.

Thank you so much for the positive evaluation. We have added or changed our descriptions for your two specific comments below.

I have the following specific questions and comments (all relatively minor) that can help further improve the manuscript.

(1) (Probably the only comment that is between major and minor): the authors make an independence assumption between the outcome model (4) and expression model (5), as explicitly stated in lines 243-244, and again in line 256. It is not clear to me what independence of two models mean exactly. It is also not clear what the consequences would be if this assumption is violated. The authors should at least make the former clear and discuss the latter.

Sorry for the confusion. We actually wanted to mean that the association analysis of models (4) and (5) are separately solved and the power of the whole analysis will be the product of the powers of them. For instance, if the power of detecting the association between expression and the trait is 0.8, and the power of detecting the association between DNA and expression is 0.7, then the overall power of the emGWAS procedure will be 0.8 x 0.7 = 0.56. Here the "independence" simply means that power of one of the models is not conditional to the other. We feel this is a natural assumption. But in the wake of the review comments, we have changed the statement in the first place (lines 243-244 in the original manuscript):

"For emGWAS, we assume that the two regression models (4) and (5) are sequentially conducted and their powers are independent of each other."

And we have also added the consequences if this assumption is violated in the second place (line 256 in the original manuscript):

“If the independence assumption is violated, i.e., the powers of these two steps are positively correlated, then the estimated power for emGWAS will be underestimated”.

(2) Lines 158-160, the authors stated “We calculate the GRM from DNA instead of expressions using the assumption that the ultimate goal is to identify genetic variants underlying expressions.” The logic here is awkward: the decision of using GRM based on genotypes is well warranted but not because the goal is to identify genetic variants underlying expression. To me, using genotypes to derive GRMs is justified because they provide reasonable and probably more accurate estimates of pairwise relationships among study participants than correlations based on predicted expression data.

Thank you for the catch. We are sorry for the awkward logic here. We have removed the related statement and instead put the reviewer’s rationale in the revised manuscript.
